# The Effect of Pretreatments and Infrared Drying on the Quality of White Radish Slices

**DOI:** 10.3390/foods15030423

**Published:** 2026-01-24

**Authors:** Małgorzata Chobot, Mariola Kozłowska, Agata Marzec, Hanna Kowalska

**Affiliations:** 1 Department of Food Engineering, Institute of Food Sciences, Warsaw University of Life Sciences, 159c Nowoursynowska St., 02-776 Warsaw, Poland; agata_marzec@sggw.edu.pl; 2 Department of Chemistry, Institute of Food Sciences, Warsaw University of Life Sciences, 159c Nowoursynowska St., 02-776 Warsaw, Poland; mariola_kozlowska@sggw.edu.pl

**Keywords:** dried vegetables, pretreatment techniques, ultrasound, PEF, spice-coating, snack texture, hygroscopicity, glass transition temperature, antioxidant activity

## Abstract

White radish is a nutritious root vegetable that provides dietary fiber, essential vitamins and minerals, and a range of bioactive compounds. This study aimed to determine how steam and microwave blanching, pulsed electric field, ultrasound, osmotic dehydration with inulin or trehalose, and coating with spices such as turmeric or sweet paprika influence the characteristics of convective infrared dried white radish slices. To assess the effect of each pretreatment, moisture content, water activity, shrinkage, density, texture, color parameters, structural characteristics (SEM and µ-CT), vapor adsorption, thermal changes, and antioxidant properties were analyzed. Osmotic dehydration with inulin most effectively limited shrinkage and color change, stabilized the microstructure, and resulted in the highest relative phenolic content and antioxidant activity (TPC, DPPH). Spice-coated samples showed low shrinkage, low hardness and breaking work, and vivid color. Furthermore, µ-CT microstructure analysis showed that these samples had the highest porosity, with those with paprika reaching 57.5%. In contrast, blanching, ultrasound, and PEF led to greater structural changes and increased hygroscopicity. Multivariate analyses confirmed the significant influence of the type of pre-treatment on the quality characteristics of the dried material. Osmotic dehydration and spice coating proved to be the most effective methods for obtaining structurally stable and visually attractive dried white radish slices with attractive bioactive compounds.

## 1. Introduction

White radish (*Raphanus sativus* L.) belongs to the Brassicaceae family. It develops an enlarged underground organ known as a hypocotyl, called the root, which is the main edible part of the plant [1]. This root, the most widely consumed part of this globally cultivated species, is typically eaten fresh, particularly in salads, or processed through marinating and cooking. Its mild pungency and crisp texture make it a popular ingredient in many culinary traditions, especially in Asian cuisines. Nutritionally, radish is valued for its high dietary fiber content (1.6 g/100 g of raw radish), low fat levels, and the presence of essential micronutrients that include B-complex vitamins, vitamin C, and minerals such as phosphorus, magnesium, calcium, zinc, iron, copper, potassium, and sodium [2]. In addition to its basic nutritional value, radish contains a variety of health-promoting phytochemicals. Among them, radish contains substantial amounts of glucosinolates, isothiocyanates, and phenolic compounds, which contribute to its antioxidant activity [3,4,5,6]. These bioactive compounds have been linked to antimicrobial, anti-inflammatory, and anticarcinogenic effects [7,8,9]. In traditional medicine, radish has long been used to treat ailments such as digestive and urinary tract disorders, liver inflammation, and cardiovascular problems [10]. Its therapeutic role in conditions like hyperlipidemia, coronary heart disease, and certain types of cancer has been highlighted [11]. Moreover, due to its low glycemic index (GI = 32), white radish is considered beneficial for individuals with diabetes, as it improves hypoglycemic activity and tissue sensitivity to insulin [12]. Despite its nutritional and functional potential, limited research has been devoted to the use of white radish as a raw material, with a focus on its valorization through drying [13,14,15,16], or other preservation techniques aimed at creating ready-to-eat snack products. Convective infrared drying has not been reported for white radish. The effects of pretreating radish with pulsed electric fields, ultrasound, or osmotic solutions made with trehalose or inulin, as well as coating with spices, have not been studied. Consequently, the changes caused by radish processing and their relationships with quality indicators, microstructural, and thermal properties remain understudied.

Drying is a method used to preserve highly perishable vegetables that have a high moisture content. Among various drying techniques, convective infrared drying has advantages over conventional hot air drying. It provides direct heat transfer to the surface of the product through electromagnetic waves, resulting in rapid moisture removal and reduced drying time [17,18]. Pretreatment methods are commonly applied prior to drying to improve moisture diffusivity and preserve the essential qualities of dried products. Various techniques have been tested on vegetables, including osmotic dehydration [19], ultrasound [20], electric field [21], edible coating [22], boiling water, microwave, and infrared blanching [23,24,25]. These methods not only support faster and more efficient moisture removal but also help retain quality attributes such as color, texture, flavor, and nutritional value. Their use is particularly relevant considering current consumer trends. There is a growing interest in shelf-stable, minimally processed vegetable products that align with plant-based diets and clean-label expectations, contributing to the expansion of the global dehydrated vegetable market [26]. In parallel, the demand for nutritious vegetable snacks with appealing sensory qualities is increasing. Recent studies have shown that vegetable-based snacks can be highly acceptable in terms of texture and flavor [27], meet nutritional expectations [28], and that both sensory quality and perceived health benefits strongly influence consumer preferences [29].

This study aimed to investigate the impact of various pretreatment methods, including pulsed electric field, ultrasound, osmotic dehydration, spice coating, and blanching techniques, on the drying parameters and quality attributes of dried white radish slices. The goal was to determine the effect of different pretreatment techniques on convective infrared drying that best support quality retention and suitability for producing dried vegetable snacks with appropriate texture and high nutritional value. To assess their effectiveness, a range of physical, functional, and sensory properties were analyzed, including color, water activity, shrinkage, macro- and micro-texture, and amorphous transition temperature.

## 2. Materials and Methods

### 2.1. Raw Materials

Fresh white radish (*Raphanus sativus* L.) roots of similar size were purchased from a local supplier. The radishes were washed thoroughly with tap water and then uniformly sliced into 3 mm-thick disks using an electric slicer (Robot Coupe CL50, Vincennes, France).

### 2.2. Pretreatment and Drying Procedure

Before drying, the white radish slices were subjected to various pretreatment methods, as described below. The abbreviations used for each treatment are provided in Table 1. Slices intended as controls were not subjected to any pretreatment and coded as CTRL.

#### 2.2.1. Blanching

Steam blanching (SB) was performed by exposing the samples to steam for 1 min, while microwave blanching (MVB) was carried out in a microwave oven at 600 W for 1 min.

#### 2.2.2. Electro-Physical Methods

Pulsed electric field treatment (PEFT) was applied using the PEFPilot™ Dual System (Elea Technology GmbH, Quakenbrück, Germany) at a specific energy input of 2 kJ. The process was conducted at a pulse frequency of 20 Hz with 79 pulses, a pulse duration of 7 μs, an electrode voltage of 24 kV, and an electric field strength of 1 kV/cm. Ultrasound treatment (UST) was performed using an ultrasonic processor (Hielscher Ultrasonics GmbH, Teltow, Germany) operating at a frequency of 24 kHz. The samples were placed on metal sieves attached to the ultrasonic device, which enabled the transmission of ultrasonic vibrations. The treatment was conducted at 100% amplitude and a 50% duty cycle for 3 min.

#### 2.2.3. Osmotic Dehydration

Two types of osmotic dehydration were used, conducted in a water bath at 55 °C for 30 min. In the first variant, the slices were immersed in a 30% inulin solution with the addition of 2% vitamin C (ODI), and in the second, in a 30% trehalose solution also containing 2% vitamin C (ODT). After immersion, the slices were gently blotted on paper to remove excess surface moisture.

#### 2.2.4. Coating

A surface coating was applied to the samples using a powdered mixture evenly distributed on both sides of the slices through a sieve. In one case, slices were coated with a blend containing 20% tapioca starch and 80% turmeric powder (TS), and in the other, with a blend of 20% tapioca starch and 80% sweet paprika powder (SPS).

#### 2.2.5. Drying

Drying was performed using a convective-infrared (IR) dryer equipped with nine PHILIPS infrared lamps, each with a power output of 175 W, arranged in three horizontal rows. The process was carried out at a constant temperature of 55 °C with an airflow velocity of 1 m/s and continued until the sample weight remained unchanged in two consecutive measurements, indicating the end of the drying process. After drying, the samples were vacuum-packed in multilayer foil pouches and heat-sealed.

**Figure 1:**
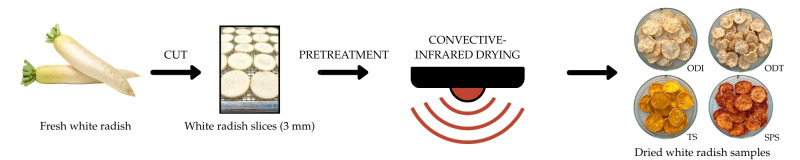
Schematic overview of the sample preparation, pretreatment, and drying process of white radish slices.

The packaged samples were stored at room temperature in a dry and dark place until further analysis. The experimental procedure is illustrated in Figure 1.

### 2.3. Analytical Methods

#### 2.3.1. Moisture Content and Water Activity

The moisture content of the dried white radish slices was determined according to the standard method using a hot air oven (SUP 65 WG, WAMED, Poland) at 105 °C for 4 h, following the guidelines of the AOAC [30]. Moisture content in relation to the mass of the samples, expressed as a percentage. Water activity was measured using a water activity meter (AQUALAB 4TE, Decagon Devices Inc., Pullman, Warsaw, WA, USA). All measurements were performed in triplicate.

#### 2.3.2. Shrinkage and Density

The shrinkage ratio of dried white radish was determined by measuring the diameter (in mm) of the same slice before (d_0_) and after drying (d). The diameters were measured using an electronic caliper with an accuracy of 0.01 cm. Measurements were performed on ten individual slices. Shrinkage was calculated using the following equation:

(1) $$Shrinkage\;(\%)=\frac{d_{0}-d}{d_{0}}\cdot 100$$

The density of samples was determined using a helium Stereopycnometer (Quantachrome, Boynton Beach, FL, USA) in triplicate for each sample, according to the method described by Qiu et al. [31] and the manufacturer’s instructions.

#### 2.3.3. Texture

Texture analysis was performed using a texture analyzer (TA HD Plus, Stable Micro Systems, Godalming, UK) equipped with a spherical probe and a 5 kg load cell. Samples were penetrated at a constant speed of 1.0 mm/s up to a distance of 7.0 mm, with a trigger force of 0.05 N. Hardness (N) was defined as the maximum force recorded on the force-displacement curve at the point of sample fracture. The breaking work (mJ) was calculated as the area under the force-displacement curve up to the maximum force. For each treatment, at least ten individual measurements were performed, and the mean value was reported.

#### 2.3.4. Color Measurement

Color parameters of dried carrot slices were measured immediately after the drying treatment using a colorimeter (Konica Minolta CR-5, Osaca, Japan). For each treatment, the assay was conducted in 10 replicates. The color was assessed in the CIE Lab* color space, where L* indicates lightness, a* represents the red-green coordinate, and b* the yellow-blue coordinate. In addition to the basic color values, chroma (C*) was calculated to provide further information on color intensity, determined using the equation:

(2) $$C^{*}=\;\sqrt{(a^{*}{)}^{2}+(b^{*}{)}^{2}}$$

To evaluate the overall color change caused by the drying process compared to the color of the raw material, the total color difference (ΔE) was calculated using the following equation:

(3) $$∆E=\sqrt{(\Delta L^{*}{)}^{2}+(\Delta a^{*}{)}^{2}+(\Delta b^{*}{)}^{2}}$$

#### 2.3.5. Vapor Adsorption Capacity (Hygroscopicity)

The samples were placed in a desiccator containing a saturated solution of sodium chloride, corresponding to a relative humidity of approximately 75% at 25 °C. Vapor adsorption was determined at predetermined time intervals of 0, 3, 6, 24, 48, 72, and 168 h. Hygroscopicity was expressed as the percentage increase in sample mass due to vapor adsorption, calculated based on the initial dry weight of the sample.

#### 2.3.6. Structure

To investigate the surface morphology of the dried white radish samples, a scanning electron microscope (Phenom XL, Thermo Fisher Scientific, Waltham, MA, USA) was used. Surface images were taken at a magnification of 200×, while internal structures were examined at 1000× and 2000× magnification. For a more detailed analysis of the internal microstructure, specifically the evaluation of porosity of the material, high-resolution X-ray microtomography was performed using a SkyScan 1272 system (Bruker, Kontich, Belgium). Volumetric reconstruction and 3D visualization of the internal structure were carried out based on cuboidal samples extracted from the central region of the dried slices. Based on the µ-CT data, quantitative 3D microstructure parameters were calculated, including total porosity, object surface area-to-volume ratio (OSVR), structure model index (SMI), and degree of anisotropy (DA).

#### 2.3.7. Thermal Analysis

The thermal properties of the dried white radish samples were analyzed using a differential scanning calorimeter with a DSC 3+ system (Mettler Toledo, Greifensee, Switzerland). Approximately 3.6 mg of the sample was placed in a sealed aluminum crucible. The analysis was conducted under a nitrogen atmosphere. The temperature program ranged from −50 °C to 150 °C, with a constant heating rate. The thermograms were used to identify thermal transitions, such as the glass transition temperature (Tg).

#### 2.3.8. Total Phenolic Content (TPC) and DPPH Radical Scavenging Activity

Dried samples were milled using an analytical mill (IKA-Werke GmbH, Staufen, Germany). Approximately 0.5 g of material was extracted with 10 mL of ethanol–water solution (80:20, *v*/*v*) for 12 h at room temperature using a multi-vortex shaker (Multi Reax, Heidolph Instruments, Schwabach, Germany). The extracts were then centrifuged at 4350 rpm for 2 min in a bench-top centrifuge (MegaStar 600, VWR, Leuven, Belgium). The supernatants were transferred into 0.2 mL PCR tubes.

The total phenolic content was determined using the Folin–Ciocalteu method [32]. An aliquot of 10 µL of the extract was mixed with 10 µL of distilled water and 40 µL of five-times-diluted Folin–Ciocalteu reagent in a 96-well plate. After 3 min, 250 µL of 7% Na_2_CO_3_ solution was added. The mixture was incubated for 1 h in the dark at room temperature. Absorbance was measured at 765 nm using a plate reader (MultiSkan Sky, Thermo Electron Co., Waltham, MA, USA) against a reagent blind. Chlorogenic acid (0–100 µg/mL) was used to prepare the calibration curve. Because the aim of the study was to compare pretreatments, TPC values were normalized to the untreated control sample (Ctrl = 100%), and results were expressed as relative (% of control). Each sample was tested in four replicates.

The antioxidant activity was determined using the DPPH radical scavenging assay, in which antioxidants present in the extract neutralize the DPPH• radical, resulting in a decrease in absorbance [33]. A stock solution of DPPH• was prepared according to [34] by dissolving 5 mg of DPPH in a 100 mL volumetric flask with methanol–water (99:1, *v*/*v*). The solution was stored in the dark at 4 °C until use.

The stock solution was then prepared with an ethanol–water solution (80:20, *v*/*v*) to obtain a working solution with an absorbance of approximately 0.7 at 515 nm. Next, 10 µL of supernatant was mixed with 250 µL of DPPH solution in a 96-well microplate. The reaction was carried out for 30 min in the dark at room temperature. Absorbance was measured at 517 nm using the same microplate reader (MultiSkan Sky, Thermo Electron Co., Waltham, MA, USA). Results were expressed relative to the DPPH control solution. Each sample was tested in four replicates. To compare the effect of pretreatments, results for both TPC and DPPH were normalized to the untreated control sample:

(4) $$Relative\;TPC/DPPH\left(\%\right)=\frac{{TPC/DPPH}_{pretreatment}}{{TPC/DPPH}_{control}}\cdot 100$$

### 2.4. Statistical Analysis

Statistical analysis of the obtained results was conducted using Statistica version 13. To determine statistical differences between the results, one-way ANOVA (*p <* 0.05) was applied, followed by post hoc analysis using the Tukey test to identify specific group differences. Additionally, Pearson correlation and principal component analysis (PCA) were conducted to explore the relationships among indicators, and the biplot technique was employed to examine similarities between data groups and samples. Repeated measures ANOVA designs were used to assess significant differences in hygroscopicity values of samples tested for 168 h.

## 3. Results and Discussion

### 3.1. Water Activity and Moisture Content

In this study, the water content and water activity of raw white radish samples were found to have high values (93.87 ± 0.08 and 0.985 ± 0.003, respectively), indicating that the fresh vegetable is susceptible to microbial deterioration. According to Park et al. [35], bacteria generally grow at water activities above 0.90, while most fungi and enzymes have a lower limit of 0.80, and most yeasts are inhibited below 0.60. In our study, all pretreatments significantly lowered water activity (Table 2), with final values ranging from 0.141 (ODI) to 0.249 (SB), suggesting that the applied pretreatment methods and drying effectively limited the potential for microbial growth by reducing the availability of free water. Except for the initial osmotic dehydration in the inulin solution, no effect of pre-treatment on the water activity of dried white radish was observed. Effective drying resulted in low moisture content across all dried samples, ranging from approx. 5.5% (PEFT) to 10.9% (ODI).

Simultaneously, no correlation was found between the decrease in water activity and water content in dried radishes. Despite the low water activity in osmotically dehydrated samples, especially those using inulin solution (ODI), the water content was higher than expected. This may be due to the presence of an osmotic substance, which could have reduced water availability for microbial growth. Similarly, low water activities were observed in samples with spice coatings. Microwave blanching proved more beneficial, and after steam treatment, water activity was higher than in the control samples. However, samples treated with a pulsed electric field had water activity similar to that of the control samples (0.246), although they had the lowest water content (approx. 5.5%).

### 3.2. Shrinkage, Density, and Texture Properties

Shrinkage reflects the structural changes and volume loss that occur during the drying process. In this study, shrinkage values ranged from 27.9% (ODI) to 49.2% (SB). The highest shrinkage was observed in the SB and PEFT samples, with approximately 50% volume reduction (Table 2), probably due to thermal softening and collapse of cellular structures during the drying process. No significant differences in shrinkage were found between the ultrasound-treated samples (46.0%) and the control or blanched samples (44.1–49.2%). Samples subjected to steam blanching and pulsed electric field treatment had the highest values of this index, reaching 49.2%. Despite partial damage to the radish tissue, these pre-treatment methods did not significantly reduce moisture content. This could be due to the specific characteristics of the raw material or uneven drying. However, Buvaneswaran et al. [36] showed that the application of ultrasound prior to drying ginger slices resulted in a lower shrinkage volume (64–65%) compared to the control and blanched samples (66–81%). Similarly, Liu et al. [37] showed that the use of ultrasound for heat pump drying of scallops resulted in reduced shrinkage and increased porosity. The lowest shrinkage was measured in the sample pretreated with osmotic dehydration using inulin (ODI), followed by samples coated with turmeric or sweet paprika (SPS and TS). For ODI samples, only about one-quarter of the original volume was lost during drying, confirming the protective role of osmotic pretreatment on structural integrity. These findings are consistent with previous studies that reported that osmotic pretreatments can significantly reduce shrinkage, as observed in apricot halves dehydrated in sucrose solution and dried by convection [38]. Dehghannya et al. [39] observed a linear relationship between moisture loss and shrinkage of Mirabelle plums. Furthermore, they indicated that, regardless of the drying rate, shrinkage can be estimated based on changes in moisture content, and even the reverse relationship can be used, i.e., determining shrinkage can indirectly indicate moisture content.

There was no significant effect of pretreatment on the density of dried radish slices (Table 2). Density ranged from 1.36 g/cm^3^ (SPS) to 1.54 g/cm^3^ (UST), reflecting the differences in porosity and structural compactness of the dried samples. In the study by Thibault et al. [40], the solid density of convectively dried apples and pears, determined by the helium pycnometer method, was 1.56 g/mL and 1.53 g/mL, respectively. Lower density of dried materials indicates higher porosity. Since root vegetables are denser than fruits, the lower density of white radishes dried with infrared convection may suggest greater porosity compared to convection-dried Mirabelle plums. The highest density in the UST sample suggests a more compact microstructure, possibly due to ultrasonic-induced structural compression or collapse. Conversely, the low density observed in SPS may be attributed to the formation of surface barriers or protective layers from spice coating, which limits shrinkage but promotes a more open and porous structure.

Mechanical properties such as hardness and breaking work varied significantly among the samples, reflecting the influence of pretreatments on the texture of dried white radish slices. The highest values were observed for the ODT sample (22.2 ± 4.6 N; 33.1 ± 6.9 mJ), indicating a very firm structure that may be undesirable for dried snack use due to its limited chewiness. Similarly, the PEFT sample exhibited relatively high hardness (15.1 ± 3.6 N), indicating a compact and rigid texture. In contrast, samples treated with spices (SPS and TS) were the softest and most brittle, with hardness values ranging from 5.3 to 5.5 N and the breaking work below 5 mJ. Similarly, the ODI sample characterized low breaking work and hardness. These samples were easier to bite and may be more acceptable to consumers seeking light, crispy, chip-like vegetable snacks. As reported by Song et al. [41], lower hardness is often associated with increased crispness, which is a desirable attribute in dried snack products. Lower density values were observed to correspond to lower hardness, breaking strength, and shrinkage values for the spice-coated samples and ODI. This is due to the spices and inulin, ingredients that filled the pores of the dried tissue and contributed to a delicate, crispier texture. Therefore, despite their lower mechanical resistance, samples like SPS and TS could provide a more favorable texture profile for ready-to-eat vegetable snacks.

### 3.3. Color

In the present study, significant differences were observed in the color parameters of white radish slices depending on the type of pretreatment applied (Table 3 and Table 4). Most of the dried samples exhibited higher lightness (L*) values compared to the raw white radish slices, indicating that the drying process generally resulted in a brighter final product appearance. Excluding the spice-coated samples, the higher absolute color difference values resulted from the increased lightness and yellowness of the microwave-blanched, ultrasonically treated, and inulin-dehydrated samples (MVB, UST, and ODI) (Table 3). However, the ODI samples showed the smallest color changes (ΔE = 11.1). Wang et al. [42] found that among several pretreatment methods of raw white radish, osmotic dehydration resulted in minimal color changes. In a recent study on drying potato strips, OD-treated samples exhibited lower browning index values compared to untreated controls and were visually described as “white-colorless” [43]. This may be desirable in the development of minimally processed food products, where visual similarity to the fresh state is valued. As shown by Elimelech et al. [44], consumers tend to associate a more natural and unaltered appearance with better quality and freshness, which positively affects their acceptance of plant-based products.

The greatest color differences compared to the raw sample (ΔE) were observed in the samples with added spices: turmeric (ΔE = 66.1) and sweet paprika (ΔE = 46.9). High ΔE values indicate that color changes are easily perceived visually, which in this case results from the intense natural pigments present in selected spices. These samples also showed the highest a* and b* values, indicating an increase in red and yellow color components. In addition, elevated chroma (C*) values confirmed the presence of a saturated and vivid color. Such intense coloration may enhance the visual appeal of dried radish snacks, as color strongly influences consumers’ expectations and decisions, and is often perceived as the primary sensory indicator of taste [45,46]. While red and yellow hues are commonly associated with tastiness [47], dried radish slices enriched with turmeric or paprika may therefore be perceived as especially attractive by consumers.

### 3.4. Vapor Adsorption Capacity

**Figure 2:**
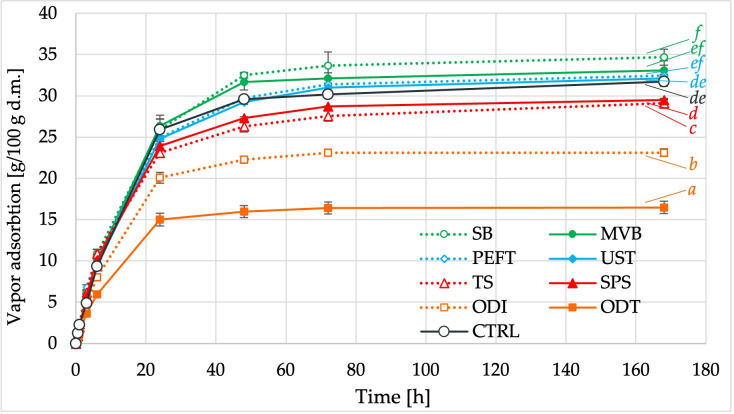
Vapor adsorption capacity of dried white radish slices subjected to different pretreatments, expressed as vapor adsorbed [g/100 g dry solid] over 168 h of storage. Points represent mean ± SD. Different letters at 168 h indicate statistically significant differences between curves (*p <* 0.05). Sample coding symbols as in Table 1.

The ability of dried white radish slices to vapor adsorption during storage varied significantly depending on the type of pretreatment used. As illustrated in Figure 2, samples subjected to blanching, electro-physical pretreatments (PEFT, UST), and untreated controls showed the highest vapor adsorption capacities, reaching over 30 g/100 g of dry solid after 72 h.

In other studies, ultrasonic pretreatment of freeze-dried strawberries has been shown to effectively reduce hygroscopicity [48]. This effect is material-dependent and may differ for other plant tissues, including white radish. Wiktor and Witrowa-Rajchert [49] further demonstrated that hygroscopic properties strongly depend on the material’s surface properties and chemical composition. The authors demonstrated that ultrasound and a pulsed electric field damaged the material’s surface, leading to an increase in the hygroscopicity of the dried carrots. In contrast, samples pretreated with osmotic dehydration absorbed significantly less water over the entire 7-day period. This limited capacity may be linked to solute impregnation and tissue compactness, which can slow water penetration. Moderate vapor adsorption was observed in the spice-enriched samples, which may reflect a balance between structural modification and the presence of added solids.

### 3.5. Structure

The macroscopic appearance, surface (200×), and cross-sectional microstructure (1000×, 2000×) of dried white radish slices subjected to different pretreatments, along with internal structure visualized with X-ray microtomography, are presented in Table 4. Based on the evaluation of the sample surfaces using SEM images, it was observed that the control samples had a relatively compact outer layer, with visible surface collapse resulting from moisture loss during the drying process. Blanched samples (SB, MVB) formed clearly distinguishable internal layers visible in cross-sectional images. This multilayered structure likely resulted from partial separation of tissue caused by heat softening, followed by compression during drying. In PEFT-treated samples, the outer cells are flattened and adhere tightly to the structures. In contrast, UST treatment resulted in a more porous structure, with greater surface roughness and visible air spaces. This finding aligns with recent reports by Lin et al. [50] and Abbaspour-Gilandeh et al. [51], who reported that ultrasound pretreatment prior to infrared drying alters the microstructure by expanding micropores, increasing the movement of free water, and supporting improved moisture transfer during drying. In slices pretreated with osmotic dehydration (ODI, ODT), cross-sectional SEM images revealed partial filling of intracellular voids, likely due to solute gain during the osmotic process. Solute uptake during the osmotic process helped stabilize the tissue matrix and reduce collapse during drying. This observation is consistent with Mari et al. [52], who demonstrated that osmotic dehydration mitigates structural damage and improves the integrity of plant tissue. Samples treated with spices (TS, SPS) showed a different surface morphology.

SEM and tomographic images indicated irregular surface layers and more flattened cross-sectional zones. These features resulted from the deposition of surface solids such as spices and starches, which may have filled intercellular spaces and limited shrinkage during drying. The internal cells appeared closely connected, resulting in a compact structure. Structural modifications were also observed in starch-coated dried pumpkin slices, where starch deposition within tissue gaps formed a surface film acting as an oxygen barrier, thereby enhancing product quality [53]. This indicates that polysaccharide deposition can influence barrier properties in vegetable samples and may contribute to the structural changes observed in dried white radish.

Several 3D microstructure parameters were measured for the tested dried plants (Table 5), including surface area-to-volume ratio (OSVR); structure model index (SMI), where an ideal value of 0 indicates a predominance of lamellar structures, while a value of 3 indicates a predominance of rod-like structures; total porosity; and degree of anisotropy (DA), where ideal values of 0 and 1 indicate total isotropy and total anisotropy, respectively. The OSVR is defined as the ratio of the pore wall surface area to the total volume of the tested sample. A high value of this parameter indicates the presence of many small-diameter pores. Samples with spice coatings, especially sweet paprika, had the highest OSVR value. However, most of the remaining samples had lower values, indicating fewer pores with larger diameters. Most dried material showed low total porosity values. The lowest porosity values were observed in samples blanched with microwaves (MVB) (approximately 0.41%) and slightly higher with steam (SB) (3.1%), with other treatments ranging from 3.41% to 6.6%. The same homogeneous group also included samples coated with turmeric (TS), but with a significantly higher porosity of 23% (Table 5). The highest porosity was found in samples coated with sweet paprika (SPS) (57%). A low porosity value reflects a higher solid content in the dried material and a lower proportion of pores. Furthermore, most of the determined 3D microstructure parameters exhibited a high standard deviation. This variability reflects heterogeneity in the microstructure, which is also confirmed by the high DA values. This likely resulted in the lack of statistically significant differences in SMI values between the different types of dried white radish samples.

### 3.6. Thermal Analysis

**Figure 3:**
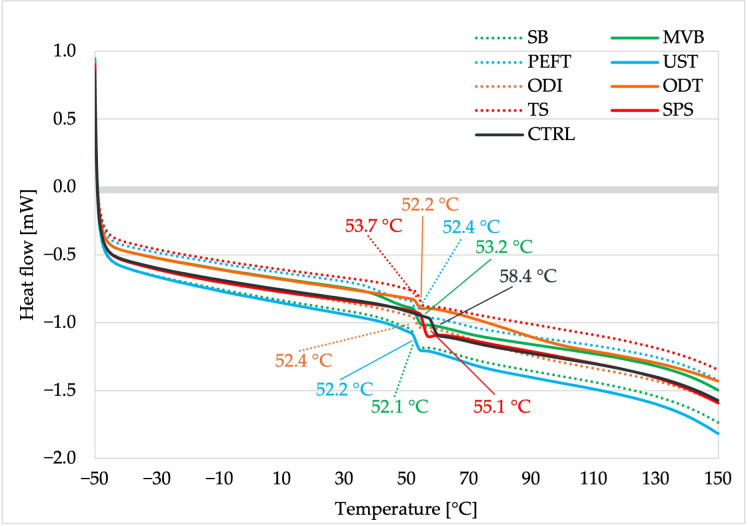
DSC thermograms of the dried white radish samples, with the glass transition temperature (Tg) indicated on the curves.

The DSC analysis revealed that all samples exhibited a similar thermal transition from the glassy (amorphous) to the rubbery state (Figure 3). The control sample had the highest glass transition temperature (Tg = 58.4 °C), whereas the pretreated samples displayed slightly lower Tg values, ranging from approximately 52.1 to 55.1 °C. Each thermogram displayed a single baseline shift characteristic of a glass transition, without additional melting or crystallization peaks. Such a pattern is typical for amorphous food matrices and has been reported for fruit powders by Caparino et al. [54]. This confirms that the pretreatments only caused moderate plasticization without changing the fundamental amorphous nature of the dried radish. The slight changes observed in Tg also correspond with the mechanism described by Liu et al. [37], who demonstrated that shrinkage during drying is closely linked to the material’s glass transition. In their work, ultrasonic pretreatment shifted the critical moisture ratio at which samples underwent glass transition and increased it by about 15% compared to the untreated control.

As a result, pretreated samples reached the glassy state earlier during drying, which minimized their volumetric shrinkage. A similar mechanism can be used to interpret the behavior of radish samples in the present study. Although the Tg values were only moderately affected by the pretreatments, even small reductions in Tg may facilitate an earlier transition to a glassy state at the applied drying temperature, thereby limiting structural collapse. Additionally, according to Joardder et al. [55], lignin, one of the primary biopolymers found in plant cell walls alongside cellulose, hemicellulose, and pectin, typically demonstrates glass transition temperature (Tg) values that range from 50 to 100 °C. The Tg values observed in this study fall within this range, suggesting that the measured transitions mainly originate from lignin-rich amorphous domains.

### 3.7. Antioxidant Activity

**Figure 4:**
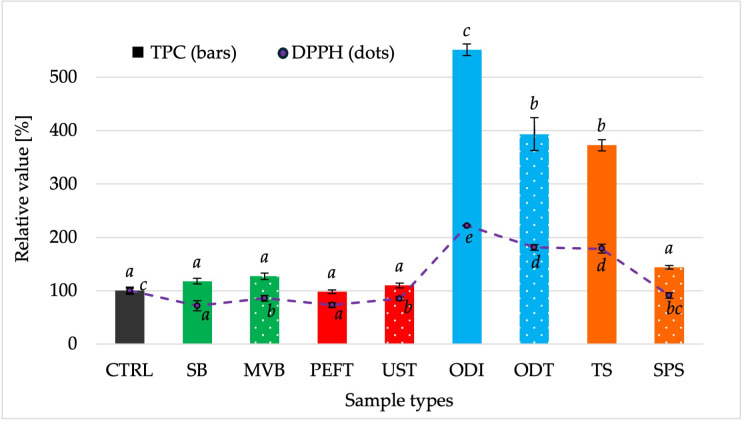
Relative total phenolic content (TPC) and radical scavenging activity content (DPPH) in white radish samples after pretreatment and drying. All values are expressed relative to the untreated control sample (CTRL). Data are presented as mean ± SD. Different letters indicate significant differences between treatments (*p <* 0.05).

As shown in Figure 4, the pretreatments had a significant impact (*p <* 0.05) on both radical scavenging activity (DPPH) and total phenolic content (TPC). The control sample was set as the baseline (100%) for comparison. Five pretreatments resulted in a reduction in antioxidant activity, with DPPH values decreasing to approximately 72% for SB and UST and remaining above 85% for MVB, PEFT, and SPS. In contrast, no statistically significant differences were detected among these treatments in terms of TPC. All samples showed comparable TPC values relative to the control, indicating that these processing conditions did not substantially affect overall phenolic availability. The strongest effects were observed for the osmotic dehydration treatments and for the turmeric coating. These samples showed higher antioxidant activity than the control, with DPPH values reaching approximately 180% for ODT and TS, and over 220% for ODI. In terms of TPC, both ODT and TS showed an approximate fourfold increase, while ODI showed a more than fivefold increase.

This suggests that the osmotic solutions used contributed to the higher antioxidant capacity observed in the treated samples, and a comparable effect was also achieved through the incorporation of antioxidants derived from turmeric. Osmotic dehydration is increasingly regarded not only as a method for partial water removal but also as an approach to enriching plant tissues with bioactive components such as polyphenols, flavonoids, and vitamin C when appropriate osmotic solutions are applied [56]. In our study, the increase in antioxidant activity observed after osmotic treatments likely reflects the combined contribution of phenolic compounds and vitamin C added. Findings from studies on dried mango indicate that adding ascorbic acid to osmotic media substantially improved vitamin C retention and, consequently, the nutritional quality of the product [57]. This effect may also be reinforced by the synergistic action of vitamin C, which supports the activity of other antioxidants and contributes to more effective free-radical scavenging effects [58,59]. A comparable outcome was observed in TS samples, where the addition of turmeric antioxidants increased both phenolic content and antioxidant capacity. This finding aligns with earlier studies that have identified turmeric as a rich source of polyphenols and curcuminoids, characterized by strong radical-scavenging and antioxidant activity [60,61,62,63].

### 3.8. Discussion of Results with Comprehensive Analysis

**Figure 5:**
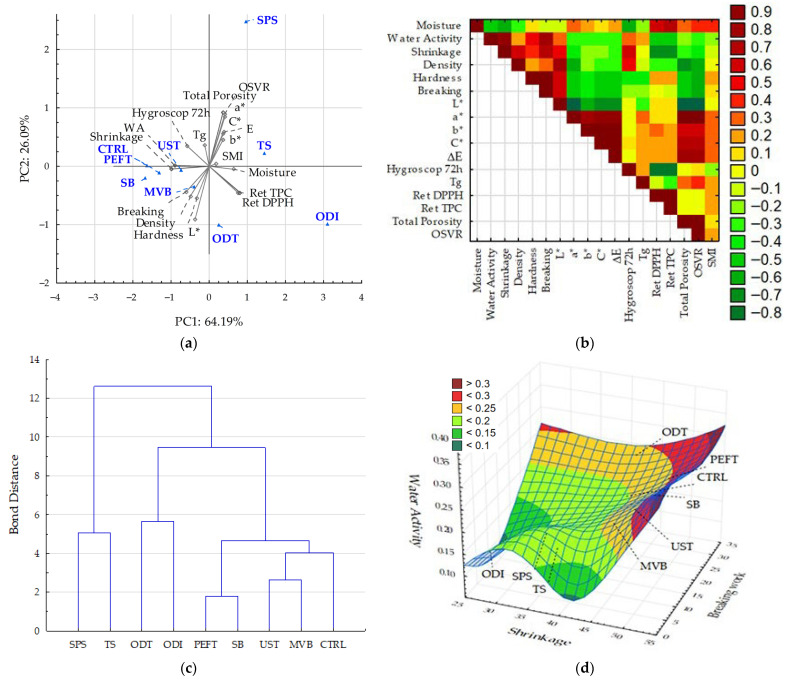
PCA and cluster analysis: (**a**) PCA loading plot of two principal components, (**b**) correlation matrix, (**c**) score plot presenting analyzed samples in terms of PC1 vs. PC2, (**d**) cluster analysis.

Principal component analysis (PCA) and hierarchical cluster analysis were used to discuss the results (Figure 5). Principal component analysis was applied to identify the variables that contributed most strongly to the differentiation of the dried radish samples (Figure 5a). The main components (PC1 and PC2) explained 90.28% of the total variability in the properties of dried white radish. PC1 mainly (64.19%) described differences in moisture behavior (water activity), shrinkage, and the retention of bioactive compounds. Polyphenol content (TPC) and DPPH antioxidant activity were located on the positive side of PC1, close to each other, indicating that these variables responded similarly, resulting in a high positive correlation (Figure 5b). High TPC and DPPH values were found in osmotically dehydrated samples (ODT and ODI) and in the turmeric-coated samples, which were positioned on the same side of PC1. Conversely, microwave-blanched samples exhibited higher density, breaking work, and hardness. These indicators, except for breaking work, did not correlate with water activity and moisture content. This indicates that the samples had similar levels of water activity and moisture content, and their properties were influenced by pretreatment. However, at lower water activity, the samples showed reduced shrinkage and a delicate texture, associated with lower breaking work (Figure 5d).

Control, ultrasonicated, and treated by PEFT samples with higher water activity were characterized by greater structural consolidation (higher density and shrinkage), increased hygroscopicity, but lower retention of bioactive compounds. Color parameters such as a*, b*, C*, and ΔE also loaded on the positive side of PC1, but in a different quadrant, indicating that they were not strongly related to compound retention. This is due to the specific composition of the osmotic solutions and the spice coatings. PC2 (26.09%) was dominated by microstructural attributes. Total porosity and OSVR were located high on the positive side of PC2 and formed a close group, indicating a strong positive correlation, as in Figure 5b. The redness value a* was in the positive part of PC2, and the brightness L* was in the opposite (negative) part, indicating that lighter samples tended to be less red but harder. In contrast, glass transition temperature and hygroscopicity showed small positive loadings on PC2, which may reflect the lower firmness of samples with reduced Tg and a higher ability to absorb moisture. The correlation heatmap (Figure 5b) relationships among moisture content, structural changes, and the retention of bioactive compounds. Moisture content showed negative correlations with density and Tg. Color parameters (a*, b*, C*, ΔE) were positively correlated with each other and negatively correlated with density and shrinkage, suggesting that color changes were greater in less structurally compact samples. TPC and DPPH retention followed the same pattern, confirming that increased structural collapse was associated with a lower preservation of bioactive compounds. A similar relationship has been reported for dried red cabbage, where greater parenchyma deformation resulted in reduced retention of phenolic compounds [64]. The authors highlighted that microstructural damage, such as cell wall disruption or a decrease in porosity, limits the ability of dried plant tissues to retain sensitive bioactive molecules. Moisture gain after 72 h correlated positively with density and shrinkage and negatively with moisture content, suggesting that more compact samples were also more prone to moisture uptake during storage.

The score plot (biplot) based on PC1 and PC2 (Figure 5a) clearly separated the samples according to the applied pretreatments. Samples located on the negative side of PC1, including ODT and ODI, were positioned in the direction of higher moisture content and retention of bioactive compounds. SPS and TS were positioned near the color vectors, showing that these attributes had a strong influence on these samples. In contrast, the UST, MVB, PEFT, SB, and CTRL samples were located on the positive side of PC1, where density, shrinkage, water activity, glass transition temperature, and moisture gain after 72 h had the strongest influence. This placement indicates that these samples underwent more pronounced structural changes. Within this group, PEFT and CTRL showed the highest PC2 scores, aligning with hardness and breaking work. UST, MVB, and SB were positioned lower on PC2, suggesting that these pretreatments influenced physical properties rather than texture.

Hierarchical cluster analysis was conducted using Ward’s method and Euclidean distance on the same set of variables used for PCA (Figure 5c). The dendrogram at a binding distance of about 6 revealed two clearly separated main clusters. The first cluster included samples CTRL, SB, MVD, PEFT, and UST. Based on the mean values of the indices, samples from this group were characterized by the highest shrinkage (46.8%) and density (1.50 g/cm^3^), but with intermediate textural characteristics compared to the samples with surface coatings, which exhibited a more delicate texture. In contrast, samples with turmeric (TS) and paprika (SPS) coatings, which were placed in the second group, exhibited significant color changes due to the surface coating of spice powder, as well as the highest porosity. The third group included samples osmotically dehydrated in a solution of inulin (ODI) and trehalose (ODT), mainly because of their lowest hygroscopicity and glass transition temperature (Tg), along with the highest polyphenol content and antioxidant activity.

## 4. Conclusions

This study demonstrates that the choice of pretreatment method has a significant impact on the final quality of convective infrared dried white radish slices. While all pretreatments ensured microbiologically safe water activity levels, their impacts on structural integrity and color varied considerably.

Osmotic treatment with an inulin solution reduced shrinkage, preserved the color of the raw material, resulted in low breaking work, and significantly improved the retention of antioxidant compounds. Slices coated with spice–starch mixtures also developed a desirable texture and vivid, attractive colors. In contrast, steam and microwave blanching, ultrasound, and pulsed electric field pretreatments, under the applied conditions, induced greater structural collapse, increased hygroscopicity, and firmer textures, making them less suitable for crispy dried products.

The results show that osmotic dehydration and spice coatings effectively transform the physicochemical properties of dried white radish slices, providing desirable qualities. Given the increasing interest in nutritious plant-based foods, these pretreatments show potential for broader applications. Future work should integrate these methods with emerging drying technologies, as well as evaluate long-term storage stability and consumer acceptance to support their practical implementation.

## Figures and Tables

**Table 1:** Pretreatment codes and their methodology.

Code	Pretreatment	Methodology
CTRL	Control	No pretreatment
SB	Steam blanching	Steam exposure for 1 min
MVB	Microwave blanching	Microwave treatment at 600 W, 1 min
PEFT	Pulsed electric field treatment	2 kJ specific energy input
UST	Ultrasound treatment	24 kHz, 50% cycle, 100% amplitude, 3 min
ODI	Osmotic dehydration (inulin)	30% inulin + 2% vitamin C, 30 min, 55 °C
ODT	Osmotic dehydration (trehalose)	30% trehalose + 2% vitamin C, 30 min, 55 °C
TS	Turmeric starch coating	80% turmeric powder 20% + tapioca starch
SPS	Sweet paprika starch coating	80% sweet paprika powder + 20% tapioca starch

**Table 2:** Selected properties of raw and dried white radish slices subjected to different pretreatments. Sample coding symbols as in Table 1.

Sample	Water Activity	Moisture Content [%]	Shrinkage [%]	Density [g/cm^3^]	Hardness [N]	Breaking Work [mJ]
CTRL	0.246 ± 0.036 ^b^	6.06 ± 1.01 ^a^	45.4 ± 4.2 ^ef^	1.48 ± 0.03 ^ab^	8.8 ± 1.7 ^abc^	15.5 ± 3.2 ^b^
SB	0.249 ± 0.027 ^b^	6.27 ± 1.92 ^a^	49.2 ± 3.4 ^f^	1.48 ± 0.03 ^ab^	9.7 ± 2.1 ^bc^	17.0 ± 5.2 ^b^
MVB	0.194 ± 0.064 ^ab^	6.67 ± 1.57 ^ab^	44.1 ± 4.1 ^de^	1.50 ± 0.03 ^ab^	11.8 ± 3.1 ^cde^	13.3 ± 5.0 ^b^
PEFT	0.246 ± 0.032 ^b^	5.46 ± 0.43 ^a^	49.1 ± 2.2 ^f^	1.49 ± 0.04 ^ab^	15.1 ± 3.6 ^e^	24.4 ± 5.6 ^c^
UST	0.206 ± 0.077 ^ab^	6.82 ± 1.14 ^ab^	46.0 ± 3.1 ^ef^	1.54 ± 0.04 ^b^	12.4 ± 3.9 ^de^	17.7 ± 6.9 ^b^
ODI	0.141 ± 0.018 ^a^	9.86 ± 1.46 ^ab^	27.9 ± 3.7 ^a^	1.44 ± 0.02 ^abc^	8.1 ± 2.4 ^ab^	3.8 ± 2.3 ^a^
ODT	0.234 ± 0.047 ^ab^	10.87 ± 0.98 ^b^	41.4 ± 4.3 ^cd^	1.42 ± 0.02 ^ac^	22.2 ± 4.6 ^f^	33.1 ± 6.9 ^d^
TS	0.171 ± 0.040 ^ab^	6.31 ± 0.27 ^a^	38.2 ± 4.4 ^bc^	1.47 ± 0.01 ^ab^	5.5 ± 0.9 ^a^	4.8 ± 2.5 ^a^
SPS	0.199 ± 0.030 ^ab^	6.26 ± 0.22 ^a^	36.5 ± 3.7 ^b^	1.36 ± 0.02 ^a^	5.3 ± 1.0 ^a^	4.8 ± 1.9 ^a^

Letters within the same column indicate statistically significant differences (*p* < 0.05).

**Table 3:** Color parameters of raw and dried white radish slices after different pretreatments. Sample coding symbols as in Table 1.

Sample	L*	a*	b*	C*	ΔE
*RAW*	64.1 ± 3.5 ^b^	−0.8 ± 0.1 ^a^	1.8 ± 0.6 ^a^	1.8 ± 0.7 ^b^	-
*CTRL*	71.0 ± 6.6 ^a^	−1.6 ± 0.3 ^ab^	12.1 ± 2.3 ^c^	12.2 ± 2.3 ^a^	13.6 ± 4.1 ^c^
*SB*	71.1 ± 6.0 ^a^	−1.8 ± 0.4 ^ab^	12.8 ± 2.6 ^cd^	12.9 ± 2.5 ^a^	14.0 ± 4.0 ^c^
*MVB*	73.3 ± 5.4 ^a^	−1.9 ± 0.6 ^ab^	13.9 ± 3.4 ^cde^	14.1 ± 3.3 ^a^	15.9 ± 4.5 ^c^
*PEFT*	70.8 ± 5.7 ^a^	−2.1 ± 0.3 ^b^	12.7 ± 2.5 ^cd^	12.9 ± 2.5 ^a^	13.7 ± 4.0 ^c^
*UST*	73.1 ± 7.5 ^a^	−0.8 ± 1.4 ^ab^	15.6 ± 4.8 ^de^	12.2 ± 4.2 ^a^	18.3 ± 3.7 ^c^
*ODI*	70.1 ± 6.6 ^a^	−1.1 ± 0.4 ^ab^	9.1 ± 2.1 ^b^	7.5 ± 2.7 ^c^	11.1 ± 3.8 ^a^
*ODT*	73.1 ± 6.9 ^a^	−0.5 ± 1.0 ^a^	15.8 ± 3.6 ^e^	12.8 ± 5.0 ^a^	17.8 ± 4.5 ^c^
*TS*	60.2 ± 4.4 ^b^	14.3 ± 3.0 ^c^	65.9 ± 3.3 ^g^	67.5 ± 3.1 ^e^	66.1 ± 2.9 ^d^
*SPS*	43.6 ± 4.2 ^c^	23.9 ± 3.6 ^d^	35.7 ± 2.6 ^f^	43.0 ± 3.2 ^d^	46.9 ± 2.3 ^b^

Letters within the same column indicate statistically significant differences (*p* < 0.05).

**Table 4:** Macroscopic and microscopic structure of dried white radish slices subjected to different pretreatments: photographic image of the dried slice, surface microstructure (SEM, 200×), cross-sectional microstructure (SEM, 1000× and 2000×), and 3D internal morphology (X-ray microtomography). Sample coding symbols as in Table 1.

Sample	Dried Slice	Surface Microstructure/Cross-Section Microstructure			Internal Structure (µ-CT)
		200x	1000×	2000×	
		100 µm	20 µm	10 µm	
CTRL	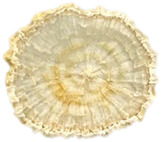	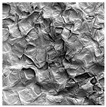	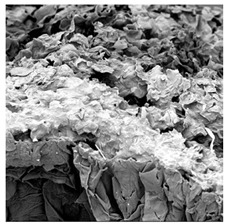	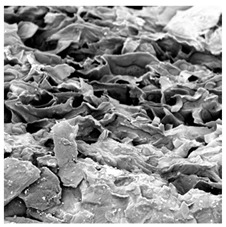	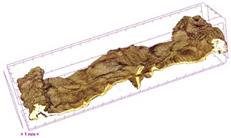
SB	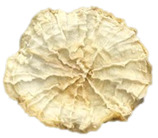	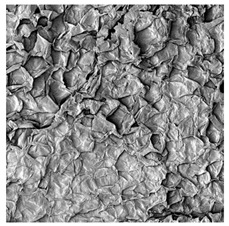	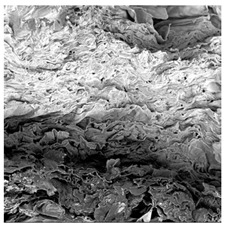	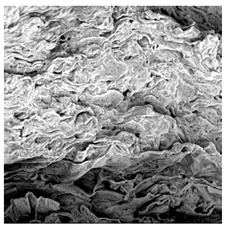	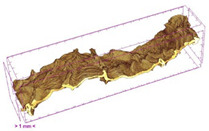
MVB	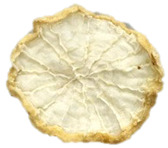	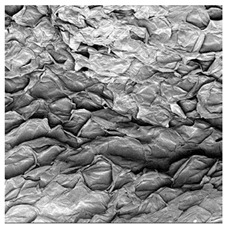	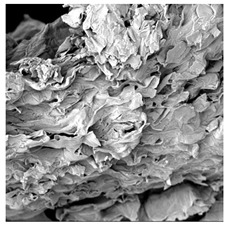	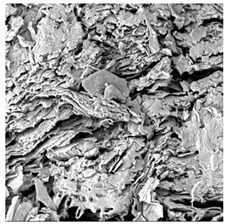	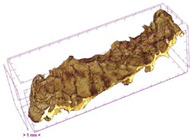
PEFT	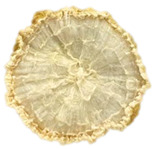	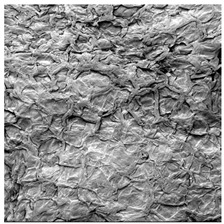	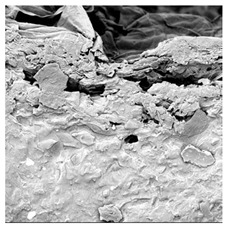	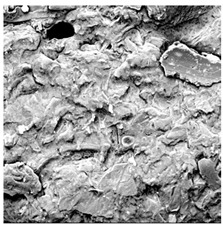	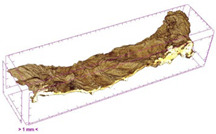
UST	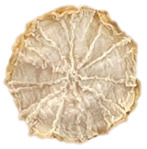	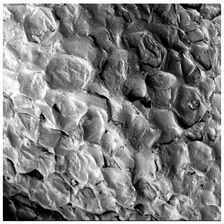	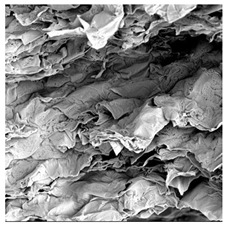	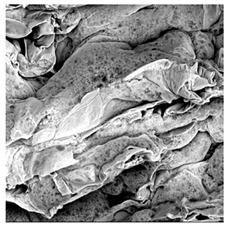	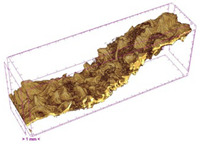
ODI	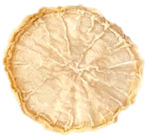	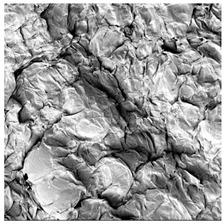	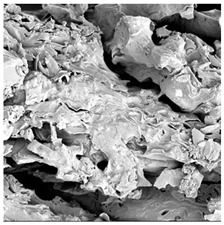	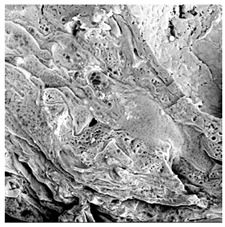	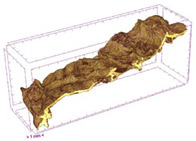
ODT	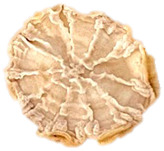	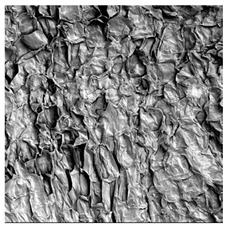	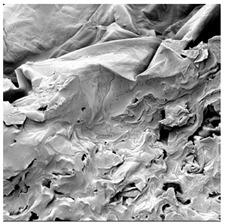	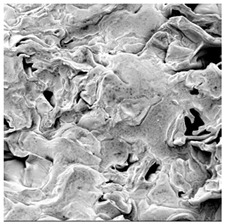	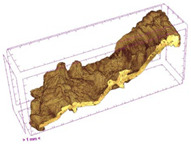
TS	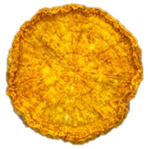	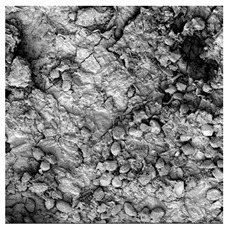	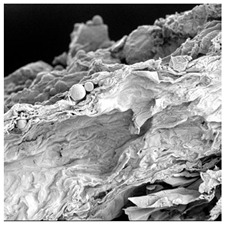	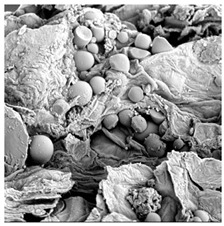	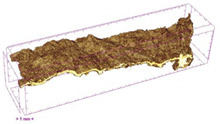
SPS	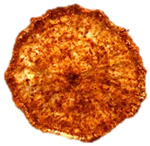	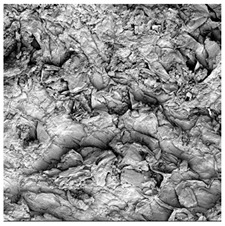	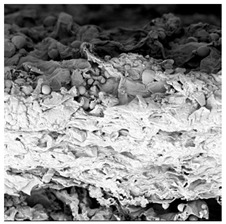	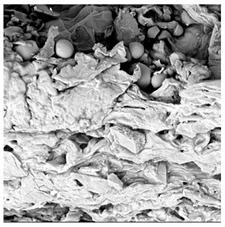	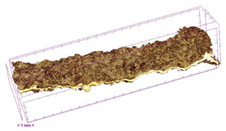

**Table 5:** Microstructure parameters of dried white radish. Statistical analysis included a one-way ANOVA at *p <* 0.05. Sample coding symbols as in Table 1.

Samples	Object Surface/ Volume Ratio (OSVR) [1/mm]	Structure Model Index (SMI)	Total Porosity [%]	Degree of Anisotropy (DA)
CTRL	45.5 ± 2.1 ^ab^	1.48 ± 0.35 ^a^	3.7 ± 3.5 ^a^	3.24 ± 0.85 ^a^
SB	37.0 ± 4.8 ^a^	1.1 ± 1.0 ^a^	3.1 ± 3.5 ^a^	35.50 ± 0.22 ^b^
MVB	38.0 ± 8.8 ^a^	2.08 ± 0.06 ^a^	0.41 ± 0.39 ^a^	16 ± 13 ^ab^
PEFT	39.0 ± 4.0 ^ab^	0.85 ± 0.28 ^a^	6.6 ± 6.0 ^a^	27 ± 26 ^ab^
UST	53 ± 28 ^ab^	1.72 ± 0.48 ^a^	5.40 ± 0.14 ^a^	23 ± 10 ^ab^
ODI	48 ± 19 ^ab^	1.20 ± 0.46 ^a^	6.4 ± 4.0 ^a^	6.6 ± 2.2 ^ab^
ODT	36.2 ± 4.2 ^a^	1.93 ± 0.31 ^a^	3.41 ± 0.24 ^a^	10.6 ± 3.9 ^ab^
TS	63 ± 28 ^ab^	1.64 ± 0.21 ^a^	23 ± 24 ^ab^	6.6 ± 2.7 ^ab^
SPS	97 ± 31 ^b^	1.65 ± 0.55 ^a^	57 ± 24 ^b^	8.6 ± 9.5 ^ab^

Different letters within the same column indicate statistically significant differences.

## Data Availability

The original contributions presented in this study are included in the article. Further inquiries can be directed to the corresponding authors.
